# Post-hatching development of mitochondrial function, organ mass and metabolic rate in two ectotherms, the American alligator (*Alligator mississippiensis*) and the common snapping turtle (*Chelydra serpentina*)

**DOI:** 10.1242/bio.017160

**Published:** 2016-03-09

**Authors:** Sarah K. G. Sirsat, Tushar S. Sirsat, Edwin R. Price, Edward M. Dzialowski

**Affiliations:** Developmental Integrative Biology Research Group, Department of Biological Sciences, University of North Texas, Denton, TX 76203, USA

**Keywords:** *Chelydra*, *Alligator*, Endothermy, Ontogeny, Mitochondria, Metabolism, Hatchling

## Abstract

The ontogeny of endothermy in birds is associated with disproportionate growth of thermogenic organs and increased mitochondrial oxidative capacity. However, no similar study has been made of the development of these traits in ectotherms. For comparison, we therefore investigated the metabolism, growth and muscle mitochondrial function in hatchlings of a turtle and a crocodilian, two ectotherms that never develop endothermy. Metabolic rate did not increase substantially in either species by 30 days post-hatching. Yolk-free body mass and heart mass did not change through 30 days in alligators and heart mass was a constant proportion of body mass, even after 1 year. Yolk-free body mass and liver mass grew 36% and 27%, respectively, in turtles during the first 30 days post-hatch. The mass-specific oxidative phosphorylation capacity of mitochondria, assessed using permeabilized muscle fibers, increased by a non-significant 47% in alligator thigh and a non-significant 50% in turtle thigh over 30 days, but did not increase in the heart. This developmental trajectory of mitochondrial function is slower and shallower than that previously observed in ducks, which demonstrate a 90% increase in mass-specific oxidative phosphorylation capacity in thigh muscles over just a few days, a 60% increase in mass-specific oxidative phosphorylation capacity of the heart over a few days, and disproportionate growth of the heart and other organs. Our data thus support the hypothesis that these developmental changes in ducks represent mechanistic drivers for attaining endothermy.

## Abstract (precis)

**Summary:** Unlike endothermic species, there is little change in skeletal and cardiac muscle mitochondrial function from the embryonic stage through the first year of life in ectothermic alligators and snapping turtles.

## INTRODUCTION

The use of metabolic heating to adjust body temperature is a fundamental feature that affects the physiology and ecology of endothermic animals. Endotherms, such as birds and mammals, tend to maintain a high and constant body temperature, and they respond to a cold challenge by increasing metabolic rate. Ectotherms, including most non-avian reptiles, tend to have a lower and more variable body temperature, and in cold environments they allow their metabolic rate to decrease and their body temperature to drop. The ability of endotherms to thermoregulate via metabolic means is not necessarily present in embryos and neonates; the capacity for endothermy develops over months in marsupial mammals (Rose et al., 1998) and over hours or weeks in birds (Whittow and Tazawa, 1991; Olson, 1992, 1994; Østnes et al., 2001; Tortosa and Castro, 2003; Sirsat et al., 2016). To maintain their high and constant body temperature, endotherms must have a high resting metabolism, high peak metabolism, adequate insulation, and the neurological ‘wiring’ that drives an endothermic response to cold. Therefore, investigation of the neonatal transition period from ectothermy to endothermy can yield insights into the mechanistic basis for endothermy.

In birds, the transition to endothermy is associated with increased capacity for O_2_ delivery via increased heart size and higher nutrient processing capacity via increased liver size (M.W. Dietz, PhD thesis, Utrecht University, 1995; Sirsat et al., 2016), disproportionate growth in skeletal muscle size (Choi et al., 1993; M.W. Dietz, PhD thesis, Utrecht University, 1995; Olson, 2001; Østnes et al., 2001), increased myofibrillar ATPase activity (Olson, 2001), and increased mass-specific catabolic capacity of muscles (Grav et al., 1988; Choi et al., 1993; Olson, 2001). At the level of the mitochondria, the capacity for oxidative phosphorylation in heart and skeletal muscle increases (Fongy et al., 2013; Sirsat et al., 2016) as does the density of mitochondria and cristae (Grav et al., 1988; Eppley and Russell, 1995) during the transition to endothermy. Additionally, there are important changes in properties of individual mitochondria during this transition (Walter and Seebacher, 2009). These findings suggest that the development of endothermy is dependent, at least in part, on this increase in capacity for oxidative phosphorylation. While there are notable changes in aerobic capacity in endotherms with development, the developmental trajectory of muscle oxidative phosphorylation capacity and associated organ changes have not previously been measured in ectotherms.

We therefore studied the development and metabolism of two ectotherms – an alligator and a turtle – during their post-hatching period. Like precocial birds, these reptiles hatch from eggs and begin unassisted feeding; unlike birds, however, they never develop endothermy. Whole-body endothermy has only arisen twice (mammals and birds) in evolutionary history, and therefore, there are few independent contrasts that can be made between ectotherms and endotherms. We chose to study American alligators (*Alligator mississippiensis*, Daudin) because they are members of the crocodilia, the sister clade to birds. Together, crocodilians and birds comprise the archosauria. The phylogenetic relationship between turtles and other reptiles has been debated (Lyson et al., 2012; Field et al., 2014), but turtles are always placed in a clade that is an outgroup to the archosauria. We therefore chose the common snapping turtle (*Chelydra serpentina*, L.) as example of an ectotherm that is no more evolutionarily related to alligators than to birds. We hypothesized that the abrupt changes in resting metabolic rate, heart size, liver size, and muscle mitochondrial oxidative capacity observed in young hatchling precocial birds are associated with the development of endothermy. Therefore, we predicted that these variables would not increase substantially in alligator or turtle hatchlings during the post-hatch period.

## RESULTS

### Whole-animal metabolic rate

In alligators, whole-animal metabolic rate did not vary significantly from the late embryonic stages through the first 30 days post hatch, but was ∼four-fold higher in yearlings (*F*_6,31_=12, *P*<0.0001; Table 1). When normalized per yolk-free body mass, metabolic rate declined significantly from Stage 25 embryos to 0 day post hatch alligators, and then did not vary significantly (*F*_6,26_=5.7, *P*=0.0006; Table 1).

**Table 1.**
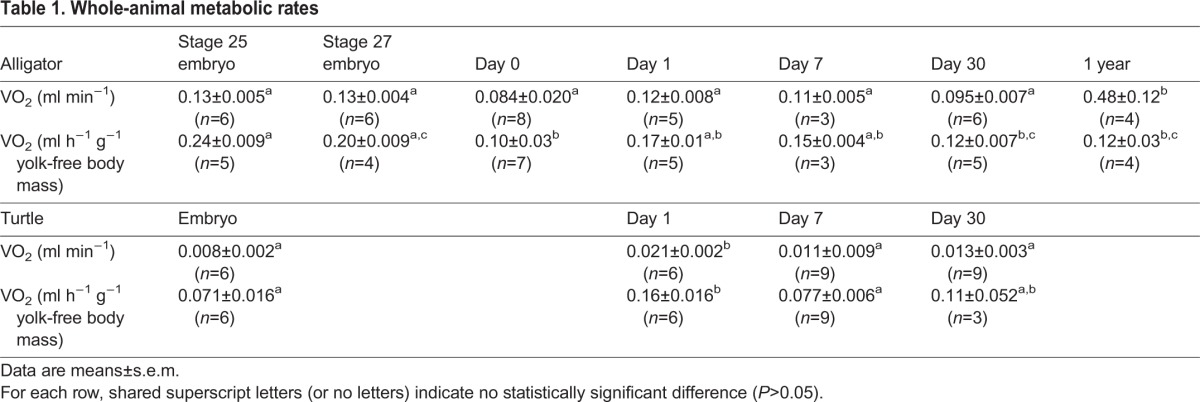
**Whole-animal metabolic rates**

In turtles, whole-animal metabolic rate peaked in 1 day post hatch turtles and then declined (*F*_3,26_=6.3, *P*=0.002; Table 1). When normalized per yolk-free body mass, metabolic rate also peaked at 1 day post hatch (*F*_3,20_=6.4, *P*=0.003; Table 1).

### Organ masses

In alligators, yolk-free body mass increased at hatching and stayed relatively constant for the first 30 days post-hatching, but was significantly elevated (five-fold) after 1 year (*F*_6,52_=493; *P*<0.0001; Table 2). Yolk sac mass diminished through development (*F*_4,43_=60, *P*<0.0001) and was not present by day 30 after hatching. Heart mass increased from stage 25 embryos to the hatch day and then remained relatively constant through 30 days after hatching, only increasing again significantly (about five-fold) after 1 year (*F*_6,53_=306, *P*<0.001; Table 2). Liver mass increased until hatching and then varied little through the first 30 days after hatching, but was five-fold higher by 1 year of age (*F*_6,53_=156, *P*<0.0001). Blood hemoglobin concentration was unchanged over post-hatching development (*F*_4,37_=1.3, *P*=0.28). Hematocrit declined during the first 30 days post hatching, and then returned to its higher level by 1 year (*F*_4,35_=3.55, *P*=0.0155; Table 2).

**Table 2.**
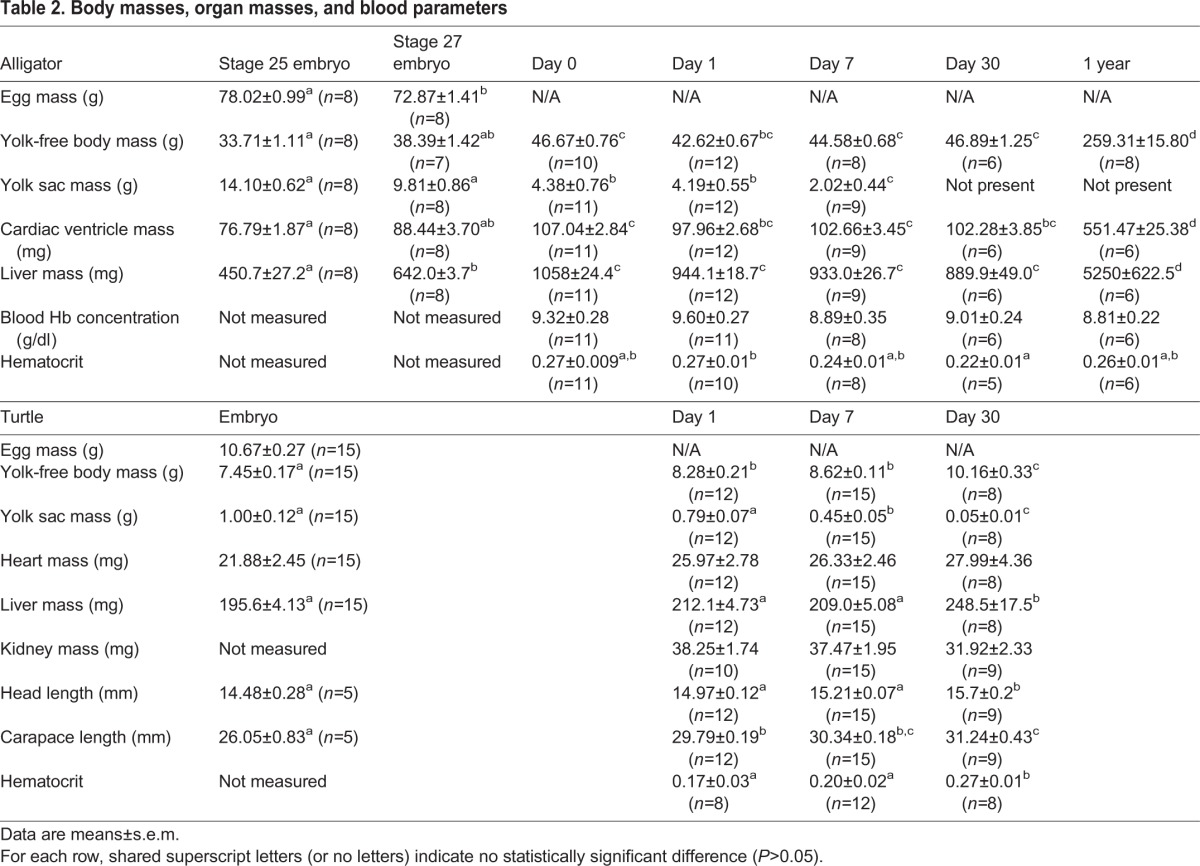
**Body masses, organ masses, and blood parameters**

In turtles, yolk-free body mass increased steadily through the first 30 days post hatch (*F*_3,46_=29.13, *P*<0.0001; Table 2), whereas yolk sac mass declined steadily (*F*_3,46_=21, *P*<0.0001). There was no significant change in heart mass during this period (*F*_3,46_=0.84, *P*=0.48). Liver mass increased only after 30 days post hatch (*F*_3,46_=7.8, *P*=0.0002). The mass of the kidneys did not change over the first 30 days after hatching (*F*_2,31_=2.4, *P*=0.10). Head length was significantly longer by the end of 30 days (*F*_3,37_=8.9, *P*<0.001), while carapace length increased throughout development (*F*_3,37_=29.8, *P*<0.0001; Table 2). Hematocrit increased after 30 days post hatch (*F*_2,25_=6.23, *P*=0.006).

### Mitochondrial function

**Fig. 1.**
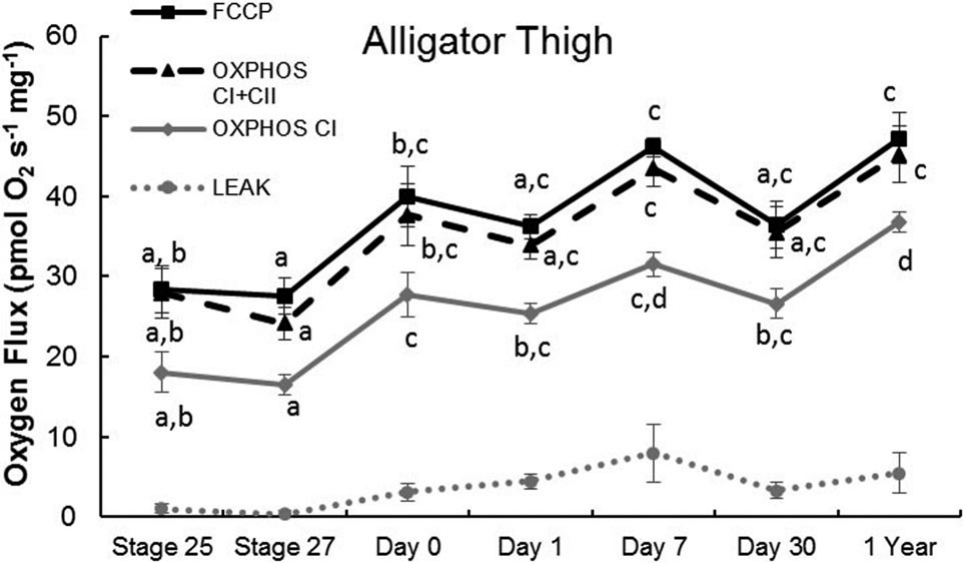
**Ontogeny of mitochondrial function in alligator thighs from late stage embryos to yearlings.** Within a state, points that share letters are not significantly different (Tukey HSD; *P*>0.05). Data are means±s.e.m. Samples sizes for each time point are equal to those in Table 3.

**Fig. 2.**
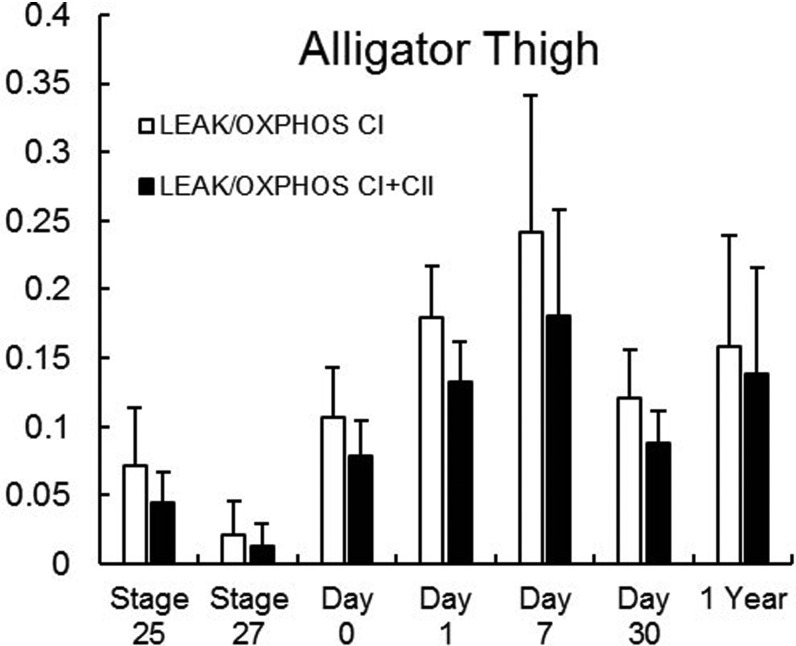
**LEAK as a proportion of OXPHOS_CI_ or OXPHOS_CI+II_ in alligator thigh muscle during development from late stage embryos to yearlings.** Data are unitless ratios and are presented as means+s.e.m. There were no significant differences among time points for either ratio (Tukey HSD; *P*>0.263 for all). Samples sizes for each time point are equal to those in Table 3.

In alligator thigh muscle, LEAK (oxygen consumption without ADP) did not vary significantly through late embryonic and post-hatching development (*F*_6,32_=1.69, *P*=0.156; Fig. 1). OXPHOS_CI_ (oxidative phosphorylation with complex I saturated) (*F*_6,32_=12.8, *P*<0.0001), OXPHOS_CI+II_ (oxidative phosphorylation with complexes I and II saturated) (*F*_6,32_=6.49, *P*<0.0002), and ETS (electron transport system) capacity (*F*_6,32_=7.96, *P*<0.001) increased from the embryo stages to hatching and further in the first year of life in alligator thigh (Fig. 1). The ratio of OXPHOS_CI_ to OXPHOS_CI+II_ increased from stage 25 embryos to yearlings (*F*_6,32_=2.21, *P*=0.0259; Table 3). FCR (flux control ratio), the ratio of OXPHOS_CI+II_ to the uncoupled ETS capacity, was unchanged during development (*F*_6,32_=1.54, *P*=0.196; Table 4). The ratio of LEAK to either OXPHOS_CI_ (*F*_6,32_=1.29, *P*=0.288) or OXPHOS_CI+II_ (*F*_6,32_=1.35, *P*=0.263) was also unchanged during development in the alligator thigh (Fig. 2).

**Table 3.**
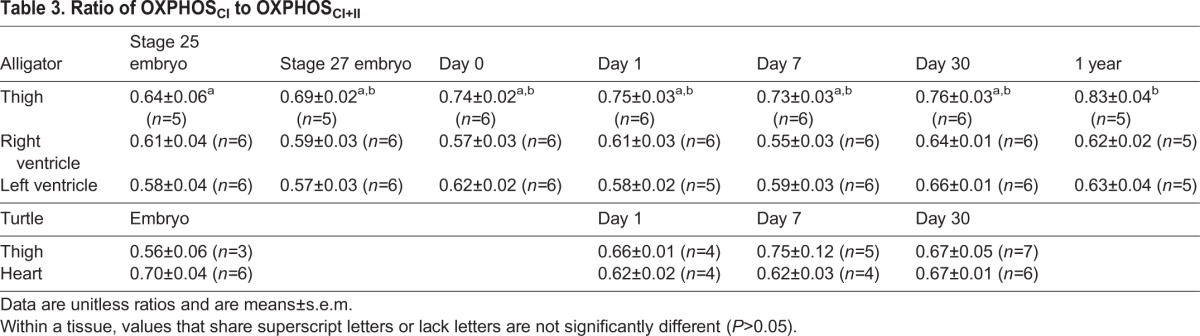
**Ratio of OXPHOS_CI_ to OXPHOS_CI+II_**

**Table 4.**
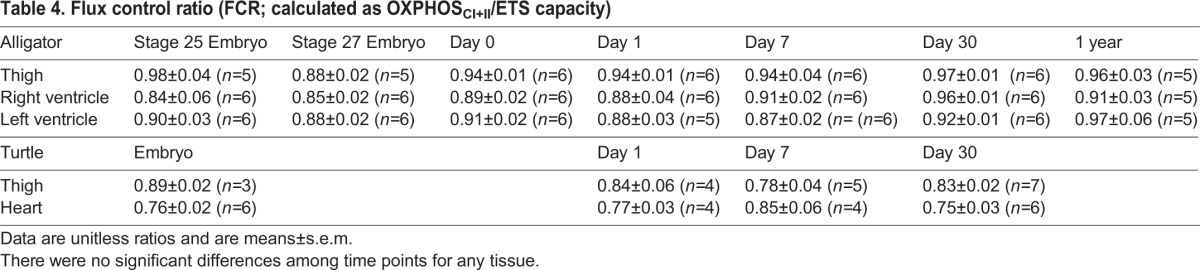
**Flux control ratio (FCR; calculated as OXPHOS_CI+II_/ETS capacity)**

**Fig. 3.**
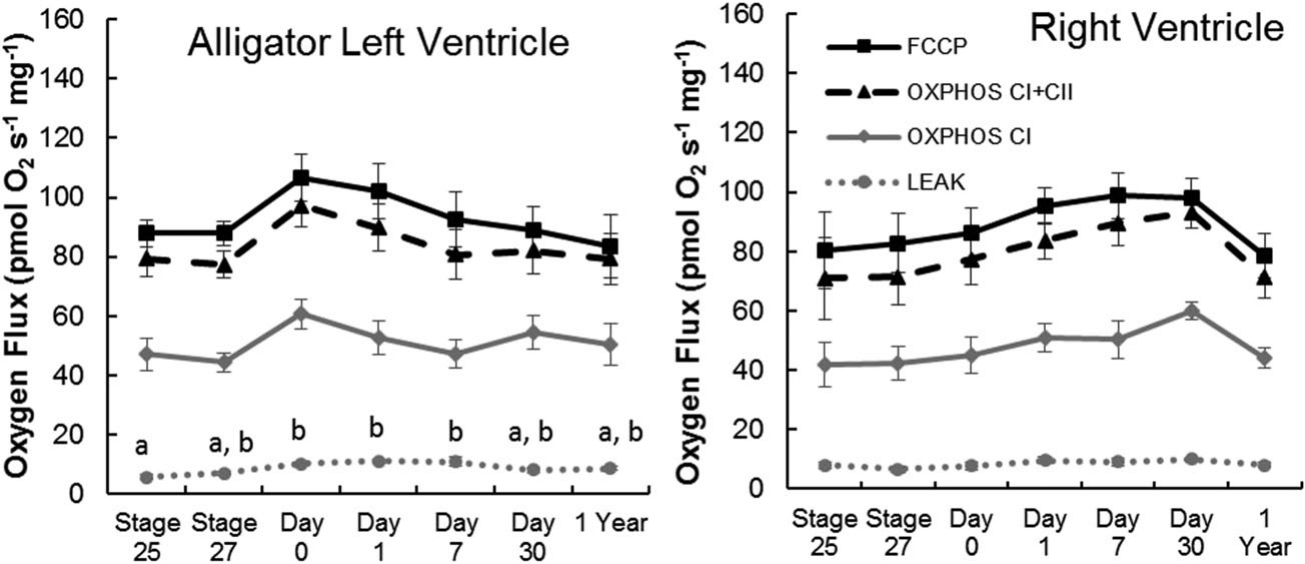
**Ontogeny of mitochondrial function in alligator heart from late stage embryos to yearlings.** Within a state, points that share letters or lack letters are not significantly different (Tukey HSD; *P*>0.05). Data are means±s.e.m. Samples sizes for each time point are equal to those in Table 3.

**Fig. 4.**
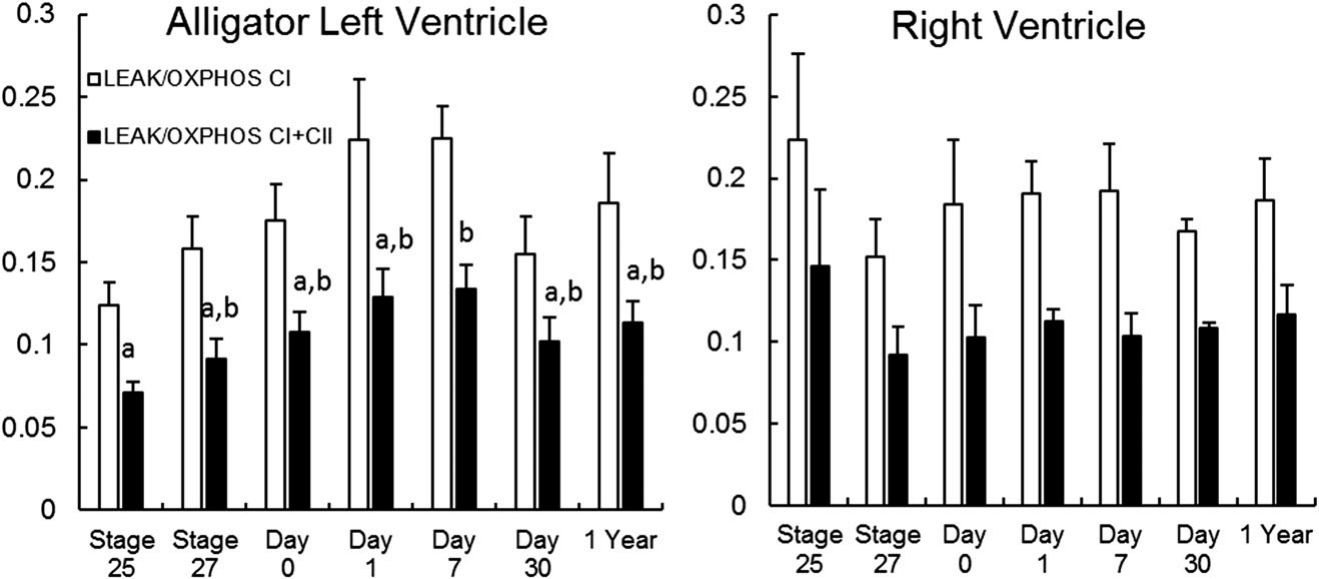
**LEAK as a proportion of OXPHOS_CI_ or OXPHOS_CI+II_ in alligator heart** **during development from late stage embryos to yearlings.** Data are unitless ratios and are presented as means+s.e.m. For a given series, bars that share letters or lack letters do not differ significantly (Tukey HSD; *P*>0.05). In the left ventricle, ANOVA detected a difference among ages in LEAK/OXPHOS_CI_, but Tukey's test did not detect a difference. Samples sizes for each timepoint are equal to those in Table 3.

In alligator hearts, there were few changes in mitochondrial function over development (Figs 3,4; Tables 3,4). In the left ventricle, LEAK was slightly elevated during the early neonate time points compared to stage 25 embryos (*F*_6,33_=4.87, *P*=0.0016; Fig. 3). Similarly, the ratio of LEAK to OXPHOS_CI+II_ was higher in the left ventricles of day 7 hatchlings compared to stage 25 embryos (*F*_6,33_=2.77, *P*=0.0272; Fig. 4). ANOVA detected a significant difference in the ratio of LEAK to OXHPOS_CI_ in left ventricle (*F*_6,33_=2.51, *P*=0.041), but Tukey's test did not detect a difference between ages. There were no other significant changes in mitochondrial function in alligator hearts (*P*>0.17 for all).

**Fig. 5.**
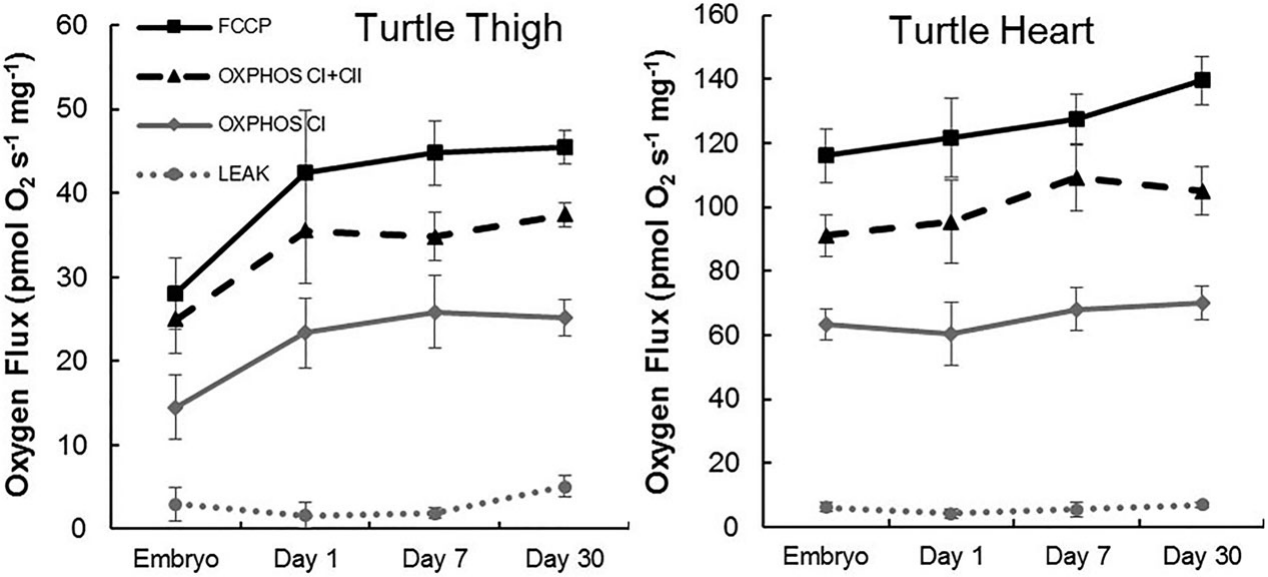
**Ontogeny of mitochondrial function in turtle thigh and heart from late stage embryos and hatchlings.** Within a state, there were no significant differences among ages (Tukey HSD; *P*>0.05). Data are means±s.e.m. Samples sizes for each timepoint are equal to those in Table 3.

**Fig. 6.**
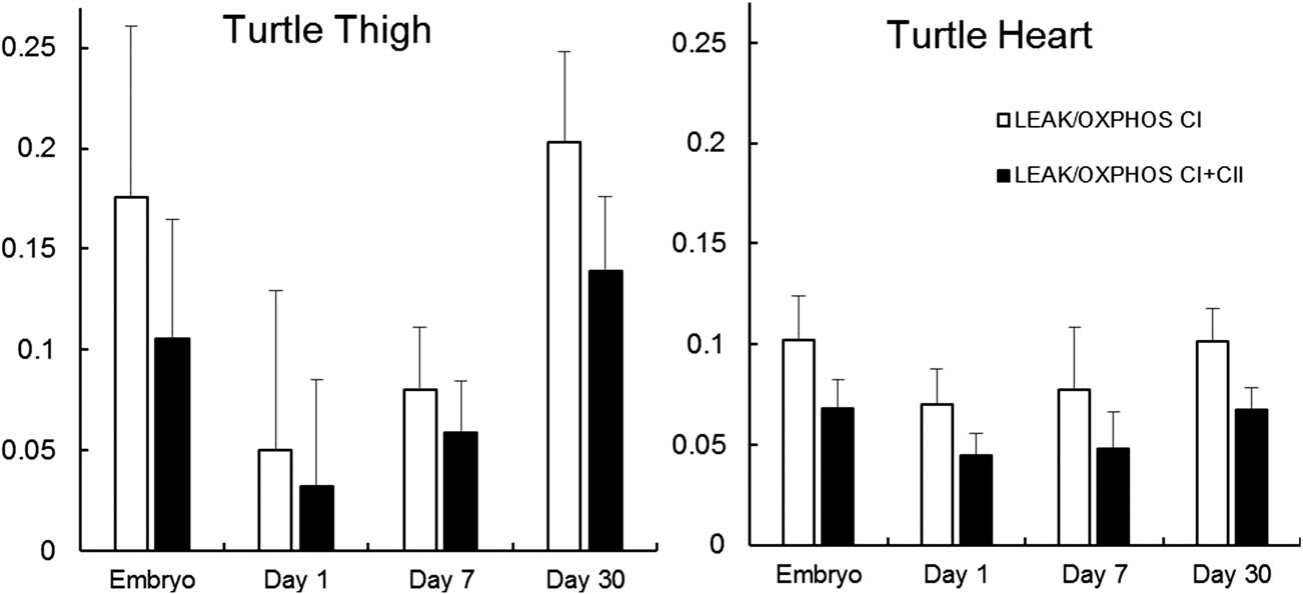
**LEAK as a proportion of OXPHOS_CI_ or OXPHOS_CI+II_ in turtle thigh and heart during development from late stage embryos to hatchlings.** Data are unitless ratios and are presented as means+s.e.m. There were no significant differences due to age for either tissue (Tukey HSD; *P*>0.05). Samples sizes for each timepoint are equal to those in Table 3.

In snapping turtles, there was a near-significant increase in the ETS capacity of thigh muscles from embryos to 30 day-old hatchlings (*F*_3,15_=2.8, *P*=0.06; Fig. 5). Otherwise, there were no significant changes in mitochondrial function among turtle ages in either thigh or heart (*P*>0.073 for all comparisons; Figs 5,6; Tables 3,4).

## DISCUSSION

### Metabolic rate

Metabolism during embryonic development in reptiles often peaks during late incubation and then plateaus or declines until hatching (Whitehead and Seymour, 1990; Thompson, 1993; Booth, 1998; Booth et al., 2000; Reid et al., 2009, but see Ackerman, 1981). Although we saw no decline in the metabolic rate of our late stage embryonic alligators, their metabolic rate may have already peaked and declined before our Stage 25 measurements. After hatching, the resting metabolic rates of turtle and crocodilian neonates may be invariant or decline somewhat over the first weeks after hatching (Jones et al., 2007; Price et al., 2007), although post-hatch increases to metabolism are also possible (Aulie and Kanui, 1995). In the current study, metabolic rate in alligators did not vary significantly over the first 30 days of development, and only increased later during the first year, by which point body mass had increased substantially. In our turtles, metabolic rate declined somewhat after 1 day post-hatch and did not increase by 30 days post-hatch. These ectotherm data contrast with the rapid increase in both overall and mass-specific metabolic rates exhibited by ducks in the hours and days after hatching (Sirsat et al., 2016).

### Organ masses

The major organs can be substantial contributors to metabolic rate, even if they represent small proportions of total body mass (Hulbert and Else, 1981; Schmidt-Nielsen, 1997). Although the heart and liver did increase in size during development, they did not do so more than the yolk-free body mass. In fact, alligator heart was a constant 0.2% of yolk-free body mass from eggs to 1-year-old animals. This result contrasts with ducks, in which heart mass, as a proportion of body mass, increased significantly from over 0.5% to nearly 1% during the transition to endothermy (Sirsat et al., 2016). Disproportionate growth of the heart in advance of endothermy has also been observed in the altricial pied flycatcher (Shilov, 1973). Similar to heart, alligator liver as a percent of body mass was approximately a constant 2% from hatching until yearlings, whereas duck livers increased from about 2% at pipping to 8% of body mass by 1 week post hatching (Sirsat et al., 2016). This contrast between ectotherms and endotherms suggests that the neonatal increases to heart and liver size in birds are associated with increased aerobic capacity and the development of endothermy, either by providing more massive thermogenic organs or through their roles in supplying the substrates of metabolism (nutrients and oxygen) to other thermogenic tissues such as skeletal muscles. Hematocrit did increase during development in turtles, which could be another mechanism for increasing oxygen delivery as an alternative to increasing heart size. However, hematocrit also was notably low in day 1 hatchlings, so this increase may merely represent maturation of the oxygen delivery system in turtles.

### Mitochondrial density and function in developing ectotherms versus endotherms

In association with their higher energetic demand, endotherms may differ from ectotherms in both the density of mitochondria and the properties of those mitochondria. We will discuss both of these in relation to our data. Because we measured OXPHOS in permeabilized fibers, our data are normalized per mg tissue; we therefore interpret our OXPHOS data as most likely representative of mitochondrial or cristae density. Other parameters such as the ratio of LEAK to OXPHOS were used to investigate the properties of those mitochondria. We focus on the thigh for comparing skeletal muscles across species.

#### Skeletal muscle

There is a clear prediction for higher mitochondrial density and oxidative capacity in endotherms compared to ectotherms, although mitochondrial oxidative capacity and whole-animal metabolic capacity do not always vary in parallel (Khan et al., 2014). Empirically, adult endotherms have higher mitochondrial density and density of oxidative enzymes than ectotherms (Else and Hulbert, 1981, 1985; Hulbert and Else, 1989). Additionally, a common response of individuals to an energetic challenge is to increase the oxidative capacity of skeletal muscle, including increases in the density of mitochondria and mitochondrial enzymes. This response can be observed in both endotherms (e.g. during bird migration; Lundgren and Kiessling, 1985; Evans et al., 1992; McFarlan et al., 2009) and ectotherms (e.g. during cold acclimation; Egginton and Sidell, 1989; Glanville and Seebacher, 2006; Guderley and Seebacher, 2011). Embryonic alligators had 49% lower OXPHOS_CI+II_ capacity per gram skeletal muscle than late-stage, pre-endothermic embryonic ducks (see data in Sirsat et al., 2016). Turtle embryos had 42% lower OXPHOS_CI+II_ than late-stage pre-endothermic embryonic ducks (data in Sirsat et al., 2016). The increase in OXPHOS during development was slight in our ectotherms, only rising a non-significant 47% in alligator thigh and a non-significant 50% in turtle thigh from late-stage embryos to 30 days post-hatching. This stands in contrast to the development of birds. OXPHOS in duck thigh increased more than 90% between the late embryonic stage and 3 days post-hatching (Sirsat et al., 2016). Thus, initial mitochondrial capacity in skeletal muscles is higher in pre-endothermic birds than ectotherms, and then increases to a much greater extent in birds during post-hatch development. This result suggests that the increase in OXPHOS in developing birds is related to the development of endothermy.

We must note that the mitochondria from ducks of that study and the turtle and alligator of the current study were measured at different temperatures (37°C for the ducks, versus 32°C for alligators and 30°C for turtles). We might try to account for this difference by applying a Q_10_ correction. This practice may be questionable due to the observation that alligators compensate for oxygen consumption, mitochondrial enzyme activity, and exercise performance when acclimated to various temperatures (Glanville and Seebacher, 2006; Guderley and Seebacher, 2011). Nonetheless, even if we assume a liberal Q_10_ of 3 (we have measured Q_10_ for OXPHOS_CI+CII_ in alligator thigh muscle to range between 2 and 2.8 when ranging between 20 and 30°C; unpublished data), alligator embryos still have ∼11% lower OXPHOS_CI+II_ than ducks, while turtle embryos had higher OXPHOS_CI+II_ than ducks using this correction. However, the large increase in OXPHOS in hatchling ducks still stands in contrast to the relatively shallow increase in OXPHOS during the development of the alligator and the turtle. This increase in avian OXPHOS capacity (measured per mg muscle) does not take into account any increase in total mass of the skeletal muscle, which grows rapidly and disproportionately in developing birds (Choi et al., 1993; Olson, 2001; Østnes et al., 2001).

Due to the higher mitochondrial OXPHOS capacity in bird muscle, which may be driven largely by higher mitochondrial density, we expected that LEAK should also be higher in birds than ectotherms and should increase more during development. Indeed, at hatching, LEAK was 3.8- and 12-fold higher in duck thigh muscle than in alligator or turtle thigh muscle, respectively (Sirsat et al., 2016). LEAK in turtle and alligator muscles changed only slightly and non-significantly, in accord with the slight or non-significant rise in OXPHOS over development. In duck thigh, LEAK increased significantly during development, again in concert with the significant and substantial rise in OXPHOS over the same period (Sirsat et al., 2016). Interestingly, there was not a significant change in the ratio of LEAK to OXPHOS in the skeletal muscle of our ectotherms or ducks, indicating that the development of endothermy in birds is associated with mitochondrial proliferation but not with changes in the proton permeability of individual mitochondria. That is to say, mitochondrial proliferation is important to supply ATP to thermogenic processes such as shivering and some types of non-shivering thermogenesis, but the muscle mitochondria apparently do not increase proton permeability as a thermogenic mechanism during the avian transition to endothermy. Nonetheless, the proportion of OXPHOS_CI_ that is LEAK tended to be lower in our alligator (2-24%) and turtle thighs (3-20%) than that found in duck thigh (9-75%; Sirsat et al., 2016). We have to caution, however, that these measurements were made at different temperatures, and that true *in vivo* proton leak will depend on other factors including membrane potential. Additionally, the coupling of proton pumping to ATP production may differ among species in other ways, such as via uncoupling proteins.

The ratio of OXPHOS_CI_ to OXPHOS_CI+II_ increased over development in the alligator thigh. Assuming that mitochondrial enzymatic capacity is optimized to the prevailing substrate available, this suggests a shift in metabolic substrate during development, perhaps from lipids toward glycolysis. Alternatively, this shift might simply reflect other changes of the electron transport system, such as differential maturation of the complexes (Piquereau et al., 2013) or different interactions of the complexes with a potentially changing mitochondrial membrane composition. Our values for FCR in ectotherms were slightly lower than those of ducks, which were closer to unity (Sirsat et al., 2016). This indicates that birds have tighter coupling of the electron transport system to the ATP synthase. Our values for OXPHOS_CI+II_ were substantially higher than observed previously in skeletal muscle of a fish (Khan et al., 2014) or a frog (Reilly et al., 2014); this may in part be due to our higher assay temperature (those experiments were conducted at 18-24°C) and choice of muscle.

#### Cardiac muscle

Many of the requirements of metabolism – oxygen and substrate delivery, and waste disposal – are tied to the circulatory system. As the primary pump for that system, the heart integrates the metabolic functions of the body. An increase in metabolic rate can therefore be expected to be associated with an increase in heart function, such as increased heart mass or contraction frequency, which in turn requires higher oxidative capacity of heart tissue. Pekin ducks during the development of endothermy demonstrated an increase in heart mass and also an increase in the mitochondrial oxidative phosphorylation capacity per gram of heart tissue (Sirsat et al., 2016). In contrast, alligators and turtles showed no change in OXPHOS capacity of the heart during the first month after hatching. Alligator heart mass did increase by one year of age, when body size had also increased, but this was not accompanied by any change in cardiac OXPHOS capacity per gram of heart. Compared to ducks (Sirsat et al., 2016), our reptiles had similar or lower OXPHOS_CI+II_ values in cardiac muscle before hatching (without taking into account any Q_10_ effects; we have measured Q_10_ values of 1.6 to 1.8 in alligator hearts; unpublished observations). Thus, as in the case of skeletal muscle, the oxidative capacity of bird hearts appears to be higher than reptiles before hatching, and then increases further during the development of endothermy. These differences are amplified even more by the increase in heart size during early post-hatch development in birds.

Leak as a proportion of OXPHOS_CI+II_ tended to be lower in our reptile hearts (∼10%) compared to birds (∼20%) (Sirsat et al., 2016), although we again caution that these measurements were made at different temperatures and possibly different membrane potentials. The ratio of OXPHOS_CI_ to OXPHOS_CI+II_ did not vary significantly over development in either alligator or turtle hearts, contrasting the increase in this ratio observed in the hearts of ducks (Sirsat et al., 2016). We interpret this as demonstrating relatively unvarying fuel mixture in reptile hearts during development. Our reptile hearts, as in skeletal muscle, tended to have lower FCRs compared to ducks (Sirsat et al., 2016), again indicating that the ETS and ATP synthase are better matched in ducks.

### Conclusions

The avian transition to endothermy is associated with rapid, disproportionate increases in muscles and other organs, and a simultaneous rapid increase in the mass-specific capacity for oxidative phosphorylation in muscles. The developmental trajectory of our non-avian reptiles, in contrast, showed minor increases in organ sizes that were proportionate to growth in body mass, and much slower and lower increases in mass-specific capacity for oxidative phosphorylation. Formal phylogenetic correction is limited, due to the small number of species examined so far and due to the few times that endothermy has evolved. However, our alligators and turtles provide two contrasts with birds, and our data thus represent the first comparative evidence for the idea that the rapid growth in organs and oxidative capacity observed in birds are direct correlates of attaining endothermic capacity.

## METHODS

### Animals and husbandry

Alligator eggs were obtained from wild nests in the Rockefeller State Wildlife Refuge and Game Preserve, Louisiana and transported to the University of North Texas (UNT) campus. Eggs were incubated in moist sphagnum moss at a temperature of 32°C so that all alligators would be the same sex (females). The incubation containers were weighed daily and misted to replace any water lost by evaporation. Upon hatching, animals were maintained in a few centimeters of water at 32°C in a constant temperature chamber and fed hatchling gator chow (Texas Farm Products Company) twice a week.

Turtle eggs were collected in North Dakota and transported to UNT. Turtle eggs were incubated in moist vermiculate at 30°C to ensure all turtles were females. Upon hatching, turtles were maintained in an inch of water at 30°C and fed hatchling gator chow twice a week. In the wild, turtle and alligator hatchlings are active after hatching and receive little or no parental assistance.

Measurements were made on alligator stage 25 and 27 embryos (staged according to (Ferguson, 1985) and representing ∼85% and 90% of incubation, respectively), as well as 0 (animals tested 0-6 h after hatching), 1, 7, and 30 days after hatching, and 1 year after hatching. Turtles were measured as embryos (on the 50th day of incubation, representing ∼95% of incubation), as well as 1, 7, and 30 days after hatching. All procedures were approved by the University of North Texas Institutional Animal Care and Use Committee.

### Whole-animal metabolic rate and morphometrics

We measured metabolic rate using flow-through respirometry in a temperature-controlled cabinet, with metabolic rate calculated as described previously (Sirsat and Dzialowski, 2016). Metabolic rate was measured at the animals' holding temperature (32°C for alligators, 30°C for turtles) after an acclimation period of at least 90 min. At the end of this period, we took averages of three 2-min recordings that were spaced over approximately 30 min. After metabolic rate measurements, the animals were anesthetized with isofluorane, and blood was obtained from the heart by cardiac puncture for later analysis. The animals were then euthanized by decapitation under anesthesia and dissected. Wet masses of the whole body and major organs were measured fresh, and thigh muscle and heart samples were taken for measurement of mitochondrial respiration (see below). In alligators, blood hemoglobin concentration was measured using Drabkin's reagent (Sigma D5941). In turtles, we measured the straight carapace length (midline from notch to notch on the most proximal and distal scutes) and the head length (measured live from the posterior end of the supraoccipital bone to the tip of snout) using calipers.

### Measurement of mitochondrial respiration in permeabilized muscle fibers

Mitochondrial respiration was measured using permeabilized fibers from alligator thigh (m. iliotibialis) and left and right ventricle tissue, and from thigh (m. iliotibialis) and whole heart from snapping turtles. Muscle fibers from thigh and heart were permeabilized according to Pesta and Gnaiger (2012). Muscle samples were placed in ice cold BIOPS solution (2.77 mM CaK_2_EGTA, 7.23 mM K_2_EGTA, 5.77 mM Na_2_ATP, 6.56 mM MgCl_2_ 6H_2_O, 20 mM taurine, 15 mM Na_2_-phosphocreatine, 20 mM imidazole, 0.5 mM dithiothreitol, 50 mM 4-morpholineethanesulfonic acid; pH 7.1) and carefully teased apart with fine-tipped forceps on ice. Tissues were then gently shaken for 30 min (20 min for cardiac samples) in 2 ml of BIOPS containing 50 µg/ml saponin at 4°C. The fibers were then washed three times for 10 min each in 2 ml of respiration assay medium (MIR05; 0.5 mM EGTA, 3 mM MgCl_2_, 60 mM lactobionic acid, 20 mM taurine, 10 mM KH_2_PO_4_, 20 mM HEPES, 110 mM sucrose, and 1 g/l bovine serum albumin; pH 7.1). Three to 5 mg of permeabilized fibers were weighed and then added to the chamber of an oxygen analyzer system (Oxygraph-2K, Oroboros Instruments, Innsbruck, Austria) with 2 ml of respiration assay medium. A hyperoxygenated environment in the respiration chamber was produced by addition of 280 units/ml catalase and 2-5 µl H_2_O_2_ (200 mM) to avoid potential limitations to O_2_ diffusion into the permeabilized fibers (Pesta and Gnaiger, 2012). Subsequent additions of substrates were used to assess various parameters of mitochondrial function. Glutamate (10 mM) and malate (2 mM) provide substrates for the electron transport system through complex I. At this stage, the lack of ADP prevents oxidative phosphorylation through ATP synthase, and thus oxygen consumption during this stage (denoted LEAK) represents the oxygen consumed due to proton slip and the leak of protons back across the inner mitochondrial membrane without any oxidative phosphorylation. ADP (5 mM) was then added to provide an oxidative substrate for ATP synthase at a non-limiting concentration. This state, denoted OXPHOS_CI_, represents the maximal rate of oxidative phosphorylation when complex I is saturated. Cytochrome C (10 µM) was then added as a test of membrane integrity. Cytochrome C cannot pass the intact outer mitochondrial membrane, and can be depleted from the inner mitochondrial membrane when mitochondria are damaged (Kuznetsov et al., 2004; Pesta and Gnaiger, 2012). Samples were therefore excluded from further analysis if the addition of cytochrome C increased the oxygen flux during OXPHOS_CI_ more than 20%. Next, succinate (20 mM) was added as a substrate for complex II. This state (OXPHOS_CI+II_) represents maximal oxidative phosphorylation capacity through the combined inputs of complexes I and II. Finally, we added FCCP (1 µM; carbonylcyanide *p*-trifluoromethoxy-phenylhydrazone), which decouples the phosphorylation of ADP from the proton pumping of the electron transport system (ETS). Because it is decoupled from phosphorylation, this measurement represents the maximum ETS capacity.

We calculated several ratios of the oxygen consumption rates in these states. The ratio of OXPHOS_CI_ to OXPHOS_CI+II_ results in an index of how much the mitochondria are ‘geared’ toward particular substrates. Complex II only receives FADH_2_ input from the citric acid cycle (the FADH_2_ produced during β-oxidation eventually transfers electrons to ubiquinone while bypassing Complexes I and II), whereas Complex I potentially receives NADH input from the citric acid cycle, β-oxidation, and glycolysis. For every acetyl CoA that enters the citric acid cycle – and thus for every input through Complex II – there were 2 NADH previously produced if the source of that acetyl CoA was glucose or exogenous lactate, 1 NADH if the source was exogenous pyruvate, and less than 1 NADH if the source was a fatty acid (Berg et al., 2002). We also calculated LEAK/OXPHOS_CI_ and LEAK/OXPHOS_CI+II_, which represent the proportions of oxygen consumption during phosphorylation that is devoted to overcoming proton leak. In reality, these are qualitative indexes and not true proportions, because the membrane potential (i.e. the proton-motive force that drives ADP phosphorylation and proton leak) will be different in the LEAK and OXPHOS states. Finally, we calculated the flux control ratio (FCR) as OXPHOS_CI+CII_/ETS Capacity. This ratio represents how well-coupled the electron transport system is to phosphorylation. Smaller FCR values indicate that there is more electron transport capacity than can be met by the quantity of ATP synthase.

### Statistical analysis

We conducted statistical analyses on metabolic rate, organ and body masses, and mitochondrial measurements for each species using R (R Core Team, 2014). For a given variable, differences among ages were tested using ANOVA with age as a factor, followed by Tukey's HSD test for multiple comparisons. When there was heterogeneity of variance (Levene's test), data were log-transformed prior to ANOVA. Sample sizes varied for each variable and age; sample sizes for each test are provided in the tables or figures in which those data are presented. *P*<0.05 was considered significant.

## References

[BIO017160C1] Ackerman R. A. (1981). Oxygen consumption by sea turtle (*Chelonia*, *Caretta*) eggs during development. *Physiol. Zool.* 54, 316-324. 10.1086/physzool.54.3.30159946

[BIO017160C2] Aulie A. and Kanui T. I. (1995). Oxygen consumption of eggs and hatchlings of the Nile crocodile (*Crocodylus niloticus*). *Comp. Biochem. Physiol. A Mol. Integr. Physiol.* 112, 99-102. 10.1016/0300-9629(95)00084-K

[BIO017160C3] Berg J. M., Tymoczko J. L. and Stryer L. (2002). *Biochemistry*, 5th edn. New York: W. H. Freeman and Company.

[BIO017160C4] Booth D. T. (1998). Effects of incubation temperature on the energetics of embryonic development and hatchling morphology in the Brisbane river turtle *Emydura signta*. *J. Comp. Physiol. B Biochem. Syst. Environ. Physiol.* 168, 399-404. 10.1007/s003600050159 9706707

[BIO017160C5] Booth D. T., Thompson M. B. and Herring S. (2000). How incubation temperature influences the physiology and growth of embryonic lizards. *J. Comp. Physiol. B Biochem. Syst. Environ. Physiol.* 170, 269-276. 10.1007/s003600000097 10935517

[BIO017160C6] Choi I.-H., Ricklefs R. E. and Shea R. E. (1993). Skeletal muscle growth, enzyme activities, and the development of thermogenesis: a comparison between altricial and precocial birds. *Physiol. Zool.* 66, 455-473. 10.1086/physzool.66.4.30163803

[BIO017160C7] Egginton S. and Sidell B. D. (1989). Thermal acclimation induces adaptive changes in subcellular structure of fish skeletal muscle. *Am. J. Physiol. Regul. Integr. Comp. Physiol.* 256, R1-R9. 10.1152/ajpregu.1989.256.1.R1 2912202

[BIO017160C8] Else P. L. and Hulbert A. J. (1981). Comparison of the “mammal machine” and the “reptile machine”: energy production. *Am. J. Physiol. Regul. Integr. Comp. Physiol.* 240, R3-R9. 10.1152/ajpregu.1981.240.1.R3 6257122

[BIO017160C9] Else P. L. and Hulbert A. J. (1985). An allometric comparison of the mitochondria of mammalian and reptilian tissues: the implications for the evolution of endothermy. *J. Comp. Physiol. B* 156, 3-11. 10.1007/BF00692920 3836230

[BIO017160C10] Eppley Z. A. and Russell B. (1995). Perinatal changes in avian muscle: implications from ultrastructure for the development of endothermy. *J. Morphol.* 225, 357-367. 10.1002/jmor.1052250307 7674307

[BIO017160C11] Evans P. R., Davidson N. C., Uttley J. D. and Evans R. D. (1992). Premigratory hypertrophy of flight muscles: an ultrastructural study. *Ornis Scand.* 23, 238-243. 10.2307/3676644

[BIO017160C12] Ferguson M. W. J. (1985). Reproductive biology and embryology of the crocodilians. In *Biology of the Reptilia: Volume 14 Development A* (ed. Gans C., Billett F. and Maderson P. F. A.), pp. 329-491. New York: John Wiley and Son.

[BIO017160C13] Field D. J., Gauthier J. A., King B. L., Pisani D., Lyson T. R. and Peterson K. J. (2014). Toward consilience in reptile phylogeny: miRNAs support an archosaur, not lepidosaur, affinity for turtles. *Evol. Dev.* 16, 189-196. 10.1111/ede.12081 24798503 PMC4215941

[BIO017160C14] Fongy A., Romestaing C., Blanc C., Lacoste-Garanger N., Rouanet J.-L., Raccurt M. and Duchamp C. (2013). Ontogeny of muscle bioenergetics in Adélie penguin chicks (*Pygoscelis adeliae*). *Am. J. Physiol. Regul. Integr. Comp. Physiol.* 305, R1065-R1075. 10.1152/ajpregu.00137.2013 24005252

[BIO017160C15] Glanville E. J. and Seebacher F. (2006). Compensation for environmental change by complementary shifts of thermal sensitivity and thermoregulatory behaviour in an ectotherm. *J. Exp. Biol.* 209, 4869-4877. 10.1242/jeb.02585 17142675

[BIO017160C16] Grav H. J., Borch-Iohnsen B., Dahl H. A., Gabrielsen G. W. and Steen J. B. (1988). Oxidative capacity of tissues contributing to thermogenesis in eider (*Somateria mollissima*) ducklings: changes associated with hatching. *J. Comp. Physiol. B* 158, 513-518. 10.1007/BF00692558 2854830

[BIO017160C17] Guderley H. and Seebacher F. (2011). Thermal acclimation, mitochondrial capacities and organ metabolic profiles in a reptile (*Alligator mississippiensis*). *J. Comp. Physiol. B* 181, 53-64. 10.1007/s00360-010-0499-1 20680297

[BIO017160C18] Hulbert A. J. and Else P. L. (1981). Comparison of the “mammal machine” and the “reptile machine”: energy use and thyroid activity. *Am. J. Physiol. Regul. Integr. Comp. Physiol.* 241, R350-R356. 10.1152/ajpregu.1981.241.5.R350 7304780

[BIO017160C19] Hulbert A. J. and Else P. L. (1989). Evolution of mammalian endothermic metabolism: mitochondrial activity and cell composition. *Am. J. Physiol. Regul. Integr. Comp. Physiol.* 256, R63-R69. 10.1152/ajpregu.1989.256.1.R63 2536249

[BIO017160C20] Jones T. T., Reina R. D., Darveau C.-A. and Lutz P. L. (2007). Ontogeny of energetics in leatherback (*Dermochelys coriacea*) and olive ridley (*Lepidochelys olivacea*) sea turtle hatchlings. *Comp. Biochem. Physiol. A Mol. Integr. Physiol.* 147, 313-322. 10.1016/j.cbpa.2006.09.013 17126576

[BIO017160C21] Khan J. R., Iftikar F. I., Herbert N. A., Gnaiger E. and Hickey A. J. R. (2014). Thermal plasticity of skeletal muscle mitochondrial activity and whole animal respiration in a common intertidal triplefin fish, *Forsterygion lapillum* (Family: Tripterygiidae). *J. Comp. Physiol. B* 184, 991-1001. 10.1007/s00360-014-0861-9 25269418

[BIO017160C22] Kuznetsov A. V., Schneeberger S., Seiler R., Brandacher G., Mark W., Steurer W., Saks V., Usson Y., Margreiter R. and Gnaiger E. (2004). Mitochondrial defects and heterogeneous cytochrome *c* release after cardiac cold ischemia and reperfusion. *Am. J. Physiol. Heart Circ. Physiol.* 286, H1633-H1641. 10.1152/ajpheart.00701.2003 14693685

[BIO017160C23] Lundgren B. O. and Kiessling K.-H. (1985). Seasonal variation in catabolic enzyme activities in breast muscle of some migratory birds. *Oecologia* 66, 468-471. 10.1007/BF00379335 28310784

[BIO017160C24] Lyson T. R., Sperling E. A., Heimberg A. M., Gauthier J. A., King B. L. and Peterson K. J. (2012). MicroRNAs support a turtle+lizard clade. *Biol. Lett.* 8, 104-107. 10.1098/rsbl.2011.0477 21775315 PMC3259949

[BIO017160C25] McFarlan J. T., Bonen A. and Guglielmo C. G. (2009). Seasonal upregulation of fatty acid transporters in flight muscles of migratory white-throated sparrows (*Zonotrichia albicollis*). *J. Exp. Biol.* 212, 2934-2940. 10.1242/jeb.031682 19717675

[BIO017160C26] Olson J. M. (1992). Growth, the development of endothermy, and the allocation of energy in red-winged blackbirds (*Agelaius phoeniceus*) during the nestling period. *Physiol. Zool.* 65, 124-152. 10.1086/physzool.65.1.30158243

[BIO017160C27] Olson J. M. (1994). The ontogeny of shivering thermogenesis in the red-winged blackbird (*Agelaius phoeniceus*). *J. Exp. Biol.* 191, 59-88. 9317339 10.1242/jeb.191.1.59

[BIO017160C28] Olson J. (2001). Ontogeny of catabolic and morphological properties of skeletal muscle of the red-winged blackbird (*Agelaius phoeniceus*). *J. Comp. Physiol. B Biochem. Syst. Environ. Physiol.* 171, 527-542. 10.1007/s003600100202 11686611

[BIO017160C29] Østnes J. E., Jenssen B. M. and Bech C. (2001). Growth and development of homeothermy in nestling European shags (*Phalacrocorax aristotelis*). *Auk* 118, 983-995. 10.1642/0004-8038(2001)118[0983:GADOHI]2.0.CO;2

[BIO017160C30] Pesta D. and Gnaiger E. (2012). High-resolution respirometry: OXPHOS protocols for human cells and permeabilized fibers from small biopsies of human muscle. In *Mitochondrial Bioenergetics: Methods and Protocols* (ed. Palmeira C. M. and Moreno A. J.), pp. 25-58. New York: Humana Press. 10.1007/978-1-61779-382-0_3 22057559

[BIO017160C31] Piquereau J., Novotova M., Garnier A., Joubert F., Veksler V. and Ventura-Clapier R. (2013). Cardiac metabolic adaptation during postnatal development. In *Cardiac Adaptations: Molecular Mechanisms* (ed. Ostadal B. and Dhalla N. S.), pp. 79-98. New York: Springer.

[BIO017160C32] Price E. R., Paladino F. V., Strohl K. P., Santidrián T. P., Klann K. and Spotila J. R. (2007). Respiration in neonate sea turtles. *Comp. Biochem. Physiol. A Mol. Integr. Physiol.* 146, 422-428. 10.1016/j.cbpa.2006.11.034 17258487 PMC1950222

[BIO017160C33] R Core Team. (2014). *R: A Language and Environment for Statistical Computing*. Vienna, Austria: R Foundation for Statistical Computing.

[BIO017160C34] Reid K. A., Margaritoulis D. and Speakman J. R. (2009). Incubation temperature and energy expenditure during development in loggerhead sea turtle embryos. *J. Exp. Mar. Biol. Ecol.* 378, 62-68. 10.1016/j.jembe.2009.07.030

[BIO017160C35] Reilly B. D., Hickey A. J. R., Cramp R. L. and Franklin C. E. (2014). Decreased hydrogen peroxide production and mitochondrial respiration in skeletal muscle but not cardiac muscle of the green-striped burrowing frog, a natural model of muscle disuse. *J. Exp. Biol.* 217, 1087-1093. 10.1242/jeb.096834 24311816

[BIO017160C36] Rose R. W., Kuswanti N. and Colquhoun E. Q. (1998). Development of endothermy in a tasmanian marsupial, *Bettongia gaimardi* and its response to cold and noradrenaline. *J. Comp. Physiol. B Biochem. Syst. Environ. Physiol.* 168, 359-363. 10.1007/s003600050155 9706706

[BIO017160C37] Schmidt-Nielsen K. (1997). *Animal Physiology: Adaptation and the Environment*, 5th edn. Cambridge: Cambridge University Press.

[BIO017160C38] Shilov I. A. (1973). *Heat Regulation in Birds (an ecological-physiological outline)*. New Delhi: Amerind Publishing Co. Pvt. Ltd.

[BIO017160C39] Sirsat T. S. and Dzialowski E. M. (2016). Ventilation changes associated with hatching and maturation of an endothermic phenotype in the Pekin duck, *Anas platyrhynchos domestica*. *Am. J. Physiol. Regul. Integr. Comp. Physiol.* 10.1152/ajpregu.00274.2015 PMC4867414 26818053

[BIO017160C40] Sirsat S. K. G., Sirsat T. S., Faber A., Duquaine A., Winnick S., Sotherland P. R. and Dzialowski E. M. (2016). Development of endothermy and concomitant increases in cardiac and skeletal muscle mitochondrial respiration in the precocial Pekin Duck (*Anas platyrhynchos domestica*) *J. Exp. Biol*. 10.1242/jeb.132282 26896549

[BIO017160C41] Thompson M. B. (1993). Oxygen consumption and energetics of development in eggs of the leatherback turtle, *Dermochelys coriacea*. *Comp. Biochem. Physiol. A Physiol.* 104, 449-453. 10.1016/0300-9629(93)90445-A

[BIO017160C42] Tortosa F. S. and Castro F. (2003). Development of thermoregulatory ability during ontogeny in the white stork *Ciconia ciconia*. *Ardeola* 50, 39-45.

[BIO017160C43] Walter I. and Seebacher F. (2009). Endothermy in birds: underlying molecular mechanisms. *J. Exp. Biol.* 212, 2328-2336. 10.1242/jeb.029009 19617425

[BIO017160C44] Whitehead P. J. and Seymour R. S. (1990). Patterns of metabolic rate in embryonic crocodilians *Crocodylus johnstoni* and *Crocodylus porosus*. *Physiol. Zool.* 63, 334-352. 10.1086/physzool.63.2.30158500

[BIO017160C45] Whittow G. C. and Tazawa H. (1991). The early development of thermoregulation in birds. *Physiol. Zool.* 64, 1371-1390. 10.1086/physzool.64.6.30158220

